# Saururus Cernuus

**Published:** 1867-07

**Authors:** D. L. Phares

**Affiliations:** Newtonia, Mi.


### ARTICLE III.

*Saururus Cernuus.* By D. L. Phares, A. M., M. D., of Newtonia, Mi.

This plant has a perennial rhizoma about three lines in diameter, one to three feet long, creeping horizontally an inch or two beneath the surface of the earth, jointed, white, tender, flexible; the internodes two to six inches long; annual stems nearly as thick as the rhizoma, one or two feet high, erect, jointed with long internodes; leaves alternate, petioled, with sheathing stipules, cordate-ovate, oblong-ovate, acuminate; spikes white, terminal, three to six inches long, nodding at apex; flowers without calyx, or corolla, white, numerous, each from the axil of a small bract, appearing in May; stamens four to eight, with long club-shaped filaments; fruit somewhat fleshy, composed of three or four partly united one or two seeded corpels, pointed with as many stigmas.

The plant grows in fresh water marshes throughout the Southern United States. The whole plant is medicinal and has a rather offensive, heavy, slightly aromatic odor and taste. It is lenitive, antispasmodic, sedative, slightly astringent. It has been much used in some parts of the country in regular as well as domestic practice, as a soothing, discutient cataplasm. It has been highly and specially recommended as a remedy to ally pain, and prevent suppuration in mammary inflammation. In these affections I have never employed it; yet I doubt not its value as a cataplasm.

But for ten or twelve years I have employed it very extensively, and with most satisfactory results in the treatment of irritations and inflammations of the kidneys, bladder, prostate gland, urethra and epididymis. It is specially indicated in all cases attended with strangury, or ardorurinæ; and when freely exhibited in warm infusion, very promptly removes the unpleasant symptoms. It is a valuable palliative in gonorrhœa and chordee; and a good vehicle for, and adjuvant to other remedies addressed to the genital urinary organs. It is not offensive to the stomach, and consequently is rarely rejected, even, when that organ is in an irritable condition; it tends rather to allay the irritation.

I would venture the suggestion, that this plant might be advantageously employed in treating some affections of the vagina, uterus and ovaries, both constitutionally, and in the former two locally. I think it might be used also beneficially in certain conditions of the nasal passages, fances, trachea, bronchia, &c.

In some parts of the country where I have introduced its use, it has become so popular, that whole plantations of it have been exhausted. A strong, hot infusion of the plant, crushed, whether dry or recent, is made. Of this, the patient may take from one to four ounces, every quarter or half hour, or only three or four times a day, according to the urgency of the symptoms or particular object had in view in its exhibition. It may often be substituted for buchu or uva ursi leaves, and in many cases, is much superior to either.

